# A comparative study of high school mathematics teachers’ audible teaching language: A student satisfaction perspective

**DOI:** 10.3389/fpsyg.2023.1108740

**Published:** 2023-02-22

**Authors:** Peijie Jiang, Xiangjun Zhang, Xiaomeng Ruan, Zirong Feng, Bin Xiong, Yanyun Jiang

**Affiliations:** ^1^School of Mathematics and Statistics, Hunan Normal University, Changsha, Hunan, China; ^2^The High School Attached to Hunan Normal University, Changsha, Hunan, China; ^3^School of Mathematical Sciences, East China Normal University, Shanghai, China; ^4^Shanghai Key Laboratory of Pure Mathematics and Mathematical Practice, Shanghai, China; ^5^School of Future Education, Beijing Normal University, Zhuhai, Guangdong, China

**Keywords:** audible teaching language, mathematics teacher, language of instruction, student satisfaction, comparative study, teaching tone, language speed

## Abstract

Teachers’ audible teaching language is essential for organizing classroom instruction. This study used a questionnaire to compare expert, skilled, and novice high school mathematics teachers’ audible teaching language from the perspective of student satisfaction. The sample was selected using a purposive sampling technique, and the participants were students from a key high school in Changsha, China. A research framework and research instrument with good reliability and validity were constructed for this study. The data were analyzed using SPSS 22.0 and AMOS 22.0. The results showed 263 valid questionnaires, good measurement model fit, and high reliability and validity of the questionnaire. It was found that: (1) students were highly satisfied with the audible teaching language of high school mathematics teachers; (2) student satisfaction with the audible teaching language of skilled, expert, and novice mathematics teachers declined in order, but there was no significant difference overall; (3) students were more satisfied with expert mathematics teachers than with novice teachers in terms of the tone and adaptability of the audible teaching language. The researchers discussed the study’s results, suggested how pre-service and post-service mathematics teachers can improve the quality of their audible teaching language, and pointed out the value and limitations of the study.

## 1. Introduction

As research in mathematics education continues to evolve, the mathematics classroom has gradually become the focus of research. Focusing on the role and functions of mathematics teachers in the classroom (Jiang et al., 2022), on what happens in the classroom (Lester, 2013), and on the influence of the classroom environment on students’ interest in learning (Liu et al., 2022) are all important for academic research. As an essential part of the mathematics classroom, teachers play a significant role in imparting knowledge. The primary way teachers transmit knowledge is through verbal expressions, including lectures, questions, and conversations, all of which are forms of audible teaching language. An audible teaching language is a form of language that transmits knowledge through sound, with pauses, soothing, and other intonations that provide information not easily conveyed through text (Carr and Kemmis, 1986). It can help learners to relate the information they hear to existing knowledge more quickly (Gaver and Gaver, 1993). Students facilitate their knowledge acquisition by listening to the teacher. Teachers can maximize the purpose of questioning and facilitate students’ learning by using verbal language to ask questions (Aziza, 2021). Productive classroom discourse is also beneficial to improve students’ performance in the classroom (Mok et al., 2022). In addition, one study found that teachers’ audible teaching language could influence classroom climate, and it indicated that a positive classroom climate could compensate for the negative effects of gender and low socioeconomic status on students’ performance in mathematics (López et al., 2022). Therefore, teachers’ classroom language is closely related to students’ learning outcomes, and teachers should use engaging and expressive language in the classroom (Ormond, 2021).

There have been several studies related to the audible teaching language for mathematics teachers. Examples include research on classroom dialog and classroom feedback. Effective classroom dialog is essential for high-quality mathematics instruction (Høynes et al., 2019), and dialog clearly relies on teachers’ audible teaching language. Some researchers have examined the characteristics of effective classroom dialog from the perspective of Chinese mathematics classrooms (Zhao et al., 2022). By examining classroom videotapes of expert and novice teachers, they found that the proportion of conversations about the basics was significantly lower, and the ratio of conversations about guessing was considerably higher in expert teachers’ classrooms compared to novice teachers. In addition, feedback in the classroom was often achieved through audible teaching language. One study investigated how 47 teachers provided feedback in 172 mathematics lessons in Norwegian junior high schools (Stovner and Klette, 2022). By analyzing the quality of feedback, the amount of feedback, and whether the feedback targeted students’ procedural skills, conceptual understanding, or engagement in mathematical practices, the researchers found that teachers spent a significant amount of time providing specific feedback, most of which was directed at procedural skills and less at conceptual feedback. Although there are some studies related to instructional language, these studies have not directly examined the audible teaching language of high school mathematics teachers and have not focused on the vocal qualities of instructional language.

In general, the language of instruction includes both spoken and silent languages. Teacher-student communication in the classroom is not only through spoken language. It is also very important for teachers to understand the non-verbal information they send and receive in the classroom (Miller, 2005). Among them, body language as a silent teaching language has attracted much attention from education researchers. Teachers’ body language, such as gestures, eyes, and expressions, plays a vital role in teaching (Woolfolk and Galloway, 1985). This role is directly reflected in that teachers should learn to use natural body movements when speaking in front of students because inappropriate actions can weaken the transmission of knowledge. Therefore, understanding and effectively using body language is also a critical teaching skill (Hale et al., 2017), and many researchers have explored teachers’ body language. For example, one study showed that teachers’ gestures not only impacted students’ learning but also on teaching itself and that different gestures had different effects (Gaythwaite, 2005; Yang et al., 2020). In various situations, gestures tend to be understood faster than using language, so gestures are considered an integral part of cognition (DeLiema et al., 2021).

Teachers’ audible language is also important. It has its drawbacks in terms of communication effectiveness (Miller, 2005) because it is non-visible and non-written, and students cannot access the information repeatedly as they can when reading a text. However, teachers’ audible language of instruction is more fundamental than body language, and the absence of audible teaching language generally means no instruction. Audible teaching language is at least as crucial as silent instructional language. Both language and the body play a vital role in the foundation of higher-order thinking, with dynamic gestures and speech making individually essential contributions to the formation of mathematical arguments (Pier et al., 2019). The teacher’s body language has to coordinate with the verbal language to convey more visual information (Ngo et al., 2022). Therefore, it is also crucial to study teachers’ audible language in mathematics classrooms. Because students serve as the focus of classroom instruction, studying the audible teaching language of high school mathematics teachers from the perspective of student satisfaction is valuable for improving mathematics learning satisfaction and teaching quality and will add new knowledge to the field of mathematics teacher education.

Several studies have compared novice teachers with expert teachers from different perspectives. One researcher compared the planning, teaching, and post-lesson reflection of three novice teachers with the three expert teachers they worked with and found that the novices’ cognitive patterns and instructional reasoning skills were not as well developed as the expert teachers (Borko and Livingston, 1989). Regarding the organization and content of mathematics teachers’ subject matter knowledge, experts categorized problems more finely and deeply, while novices had more horizontal, independent categorization systems (Leinhardt and Smith, 1985). A comparative study of the language of instruction of new and veteran mathematics teachers, on the other hand, noted that novice teachers used too much instructional language to teach knowledge, while veteran teachers were good at using instructional language to guide students’ thinking (Ye et al., 2015). These studies suggest that expert teachers are more adept than novice teachers. Therefore, in this study, we hypothesized that expert teachers were more competent than novice teachers in using audible teaching language and that the quality of teachers’ audible teaching language could be improved through appropriate training. Learning how expert teachers use the language helped novice and pre-service teachers improve their language ability.

## 2. Overview of the literature

### 2.1. Audible teaching language and its research

An audible language is a form of language that is communicated through sound and can help learners connect the information they hear with existing knowledge more quickly (Gaver and Gaver, 1993). Pauses, soothing, and other intonations provide information that is not easily conveyed through text (Carr and Kemmis, 1986). The perception and production of sound are fundamental to human cognition and behavior (Bautista and Roth, 2012). Further, the study of language use can provide insight into popular discussion topics among mathematics teachers and mathematics educators.

Audible teaching language is one of the ways to express the language of instruction. The language of instruction is logical, clear, dominant, humorous, authoritative, and developmental. Based on these characteristics, concise, logical language enhances students’ attention, leading to improved student performance (Cogan, 1958), while teachers’ clarity, expressiveness, and delivery are significantly correlated and influence students’ evaluations (Solomon et al., 1964). The language of instruction is crucial to student development (Wasik and Hindman, 2011). Some researchers have suggested that a teacher’s speaking can help students improve their comprehension (Franke et al., 2009). Teachers’ audible teaching language is part of classroom discourse, and there has been some research on classroom discourse. The teacher’s audible teaching language makes classroom dialog possible. Classroom discourse includes teacher discourse, student discourse, silence, and discussion (Jiang et al., 2018). In general, teachers’ discourse dominates classroom conversations, is authoritative, contributes to the transmission of knowledge in the classroom, and influences the structure of authority (Scott, 1998; Ng et al., 2021) and classroom effectiveness (Guo and Xia, 2021), and humorous language contributes to student learning (Do et al., 2022).

Classroom discourse is a significant component of the classroom and carries a great deal of helpful information that plays an essential role in learning (Grifenhagen and Barnes, 2022). Properly managed classroom discourse can allow students to develop their understanding and help them benefit from the ideas of their peers and teachers (Wang et al., 2014).

Classroom dialog can be divided into conversational and questioning types. On the one hand, students learn not in isolation but through dialog (Lee and Kinzie, 2012). Students’ engagement in dialogic discourse, such as questioning and connecting ideas, contributes to developing active, analytical, and personal thoughts (Scott, 1998). On the other hand, teacher questioning is a distinctive feature of classroom talk, and focusing on questioning practices helps us better understand the role of teacher questioning in scaffolding instruction. Chin (2007) explored how teachers used questions in classroom discourse to support students’ thinking and help them construct knowledge.

The audible teaching language is the teaching language carried by sound. From the above analysis, teachers’ audible teaching language is fundamental. Still, there are few achievements in studying teachers’ audible teaching language behavior, and further research is needed.

### 2.2. Comparison of expert and novice mathematics teachers

It is common to distinguish between expert and novice teachers regarding educational and teaching experience and theory knowledge (Sternberg and Horvath, 1995). Cai et al. (2022) treated mathematics teachers with more than 25 years of teaching experience as expert mathematics teachers. Some researchers referred to teachers with an average of 22 years of teaching experience as expert teachers and those with an average of 3 years of teaching experience as novice teachers (Zhao et al., 2022). In China, because there are strict professional standards for evaluating teacher titles, the titles can broadly reflect the professional competence of teachers. In this study, teachers were classified regarding their titles and years of teaching experience. Novice teachers were junior teachers who had taught for less than 5 years. Expert teachers were intermediate and advanced teachers who had taught for over 10 years. Those who have been teaching for more than 5 years and less than 10 years were classified as skilled teachers, regardless of their job title.

There is a richer body of research comparing expert and novice mathematics teachers. About a decade ago, a researcher explored the attention to classroom events of 10 experts and 10 novice teachers in China. This researcher noted that expert teachers focused more on developing students’ mathematical thinking and higher-order thinking, as well as the coherence of students’ mathematical knowledge, than novice teachers (Huang and Li, 2012). Researchers are still exploring the topic of teacher attention. One study highlighted the cultural dependence on the development of expertise in teacher attention by comparing empirical knowledge of the development of teacher attention from the novice level to the expert level (Bastian et al., 2022). Other researchers have explored more specific issues, such as how secondary school teachers and students impose personal structures on fractional expressions and equations, noting that expert teachers construct a particular fractional expression in various ways (Ruede, 2013).

More recently, some researchers have elaborated on how classroom expertise affects visual perception and mental interpretation by comparing expert and novice teachers’ knowledge levels and their decisions to act on classroom events that help clarify differences in teachers’ perceptions and representations of events (Wolff et al., 2021). Other researchers examined how expert and novice (pre-service) teachers completed mathematical modeling tasks, respectively, and how they noticed the written work of student thinking completed in response to mathematical modeling tasks. Almost all expert mathematics teachers responded by asking questions, while about one-third of pre-service mathematics teachers directly corrected students’ errors, and another third pointed out errors but did not correct them (Cai et al., 2022).

Overall, studies comparing expert and novice mathematics teachers have been numerous and have covered a wide range of topics. However, there have been few studies on the audible teaching language of expert and novice mathematics teachers. The comparative studies that have been conducted on mathematics teachers’ instructional language are based on classroom video analyzes, and the instructional language in these studies also includes, for example, body language (Ye et al., 2015) and does not specifically examine audible teaching language. Studying the differences in the audible teaching language of expert and novice mathematics teachers from the perspective of student satisfaction is theoretically and practically necessary.

### 2.3. Student satisfaction and its evaluation

Student satisfaction is the psychological feeling of satisfaction or dissatisfaction students have when they compare their perceived effectiveness with their desired effectiveness throughout instruction. As addressed in this paper, student satisfaction refers to the extent to which students feel satisfied with the mathematics teacher’s audible teaching language. Various factors influence student satisfaction, and we need to distinguish among them to determine their impact on the provision of quality education. Recognizing the specific factors affecting student satisfaction will help us develop strategies to enhance student’s learning experience and improve the education provided (Cheng, 2016, pp. 33–45).

Student satisfaction is considered a rather important aspect of educational strategy, and there is a direct, positive, and significant relationship between the quality of education and student satisfaction (Meštrović, 2017). Specifically, student satisfaction effectively influences learning ability (Panyajamorn et al., 2018). Given the influence of student satisfaction on the quality of education, student satisfaction is considered an indicator when assessing instruction content and the level of importance of mathematics subjects (Carlos Ramirez-Cruz et al., 2018). Factors that influence student satisfaction have been widely discussed, including students’ learning styles (Kim, 2021; Mahir et al., 2021), gender, students’ self-efficacy, teachers’ teaching methods and tools (Gudelj et al., 2021), and instructional materials (Lee, 2014).

Students’ perception of the classroom learning environment measures student satisfaction. Teacher professionalism affects students’ attitudes toward mathematics lessons and perceptions of classroom climate, ultimately affecting students’ academic performance and overall satisfaction with the curriculum (Suh et al., 2018). Teacher professional development should include improving teachers’ language of instruction. Teachers’ classroom discourse can reflect teachers’ audible teaching language level to some extent, which is an essential component of the classroom environment. The teacher’s central role is to guide and sustain the conversation in the desired direction related to the learning objectives and to reduce gaps in students’ performance on the intended learning objectives (Kayima and Mkimbili, 2021).

These studies point out that classroom discussion and dialog are essential avenues of teacher-student interaction, and learning satisfaction is directly influenced by learner interaction, perceived ease of use, and academic performance (Nagy, 2018). How teachers initiate and facilitate discussions when students respond essentially determines classroom interactions (Khoza and Msimanga, 2021). Therefore, facilitating discussions in the mathematics classroom effectively improves students’ thinking, reasoning, and problem-solving skills to support their mathematics learning and improve their learning ability and student satisfaction.

The above analysis revealed that student satisfaction is vital for improving the quality of education and that teacher quality is a crucial determinant of educational quality. Promoting teachers’ language skills can help to create a positive classroom environment, promote good student-teacher interaction, and increase student satisfaction. Previous research has focused more on the content of the language of instruction and less on the vocal characteristics of the teacher’s language. This study helps to advance research in this area.

## 3. Purpose of study

To understand students’ satisfaction with current high school mathematics teachers’ audible teaching language and to recognize the difference between expert and novice teachers’ use of audible teaching language, this study focused on answering the following questions:

1. How satisfied are students with the audible teaching language used by high school mathematics teachers in their classrooms?
2. What are the similarities and differences in the audible teaching language used by expert, skilled, and novice high school mathematics teachers in their classrooms?

## 4. Methods and materials

### 4.1. Framework and tools

Research frameworks and instruments on teachers’ audible teaching language are unavailable, but there are several scales for evaluating instructional language. Teachers’ discourse actions include engaging in reflective discourse, responding to prior student discourse with neutral restatements, and exploring students’ dominant ideas (Soysal and Yilmaz-Tuzun, 2021). Bekiari et al. (2006) focused on the affective expression dimension of teachers’ instructional language, examined students’ perceptions of teachers’ verbal aggression, and investigated students’ perceptions of learning activities (doing homework) and the fun nature of the school environment using a classroom satisfaction scale. Smart and Marshall (2013) proposed a structure for measuring instructional language that consisted of questioning level, question complexity, questioning ecology, patterns of engagement, and classroom interaction. The questioning level examines whether the teacher’s classroom language is asking questions to students at different levels, and question complexity focuses on whether the questions are focused on one correct answer. In a comparative study of secondary school biology teachers’ audible teaching language, researchers constructed a measurement framework that included both form and content elements (Kong, 2018). The form element includes volume, tone, and speech rate; the content element includes imagery, interest, science, conciseness, illumination, relevance, coherence, and education. The framework is the result of the Delphi method, used by eight experts and researchers to discuss the proposed indicators and the weighting and analysis of the proposed indicators, combined with the validation factor analysis after repeated revisions and discussions to form a particular reference value. However, this framework has a crossover between secondary indicators, such as correlation and coherence. Therefore, it is necessary to redesign the survey analysis framework to investigate better and analyze the audible teaching language of high school mathematics teachers from the perspective of student satisfaction. In the absence of instruments to measure teachers’ audible teaching language, the researchers developed an instrument based on the work of Kong (2018).

This study first constructed a preliminary framework based on the existing literature and the practical experiences of two skilled high school mathematics teachers. Then the basic framework (framework for analyzing high school mathematics teachers’ satisfaction with the audible teaching language) was determined using the Delphi method based on the preliminary framework, as shown in Table 1.

**Table 1:** Framework for analyzing audible teaching language.

Attribute	Indicators	Standard	Items
Sound	Volume	Appropriate loudness of sound.	t1-t2
	Tone	Appropriate use of repetition, stress, and pauses.	t3-t5
	Speed	The speed of speech should be varied.	t6-t8
	Quality	Harmonious sound and a beautiful-sounding voice.	t9-t11
Content	Figurativeness	Flexible use of similes and metaphors to teach.	t12-t14
	Interesting	The teaching is humorous and interesting.	t15-t17
	Scientificity	The teaching language is strict and standardized, with proper use of terminology.	t18-t20
	Conciseness	Concise teaching without complicated language.	t21-t23
	Inspiring	Mobilize students’ thinking in all aspects.	t24-t26
	Feedback	Provide timely feedback during teaching activities.	t27-t29
	Sincerity	Sincere and authentic feelings in teaching.	t30-t32
	Adaptability	Adapt to the needs of students’ psychology.	t33-t35

In the framework, the audible teaching language of high school mathematics teachers contains both *Sound* and *Content* attributes. The observed indicators of the *Sound* attribute are *Volume*, *Tone*, *Speed*, and *Quality*. The observed indicators of the *Content* attribute include *Figurativeness*, *Interesting*, *Scientificity*, *Conciseness*, *Inspiring*, *Feedback*, *Sincerity*, and *Adaptability*. The judgment criteria of each refined index are also listed in the table. To observe the exogenous variables, each was measured by several synonymous but differently formulated items. The average value of the synonymous items was taken as the observed value of the exogenous variable.

For example, in the case of *Volume*, “The teacher’s voice level is appropriate” and “The teacher can adjust the voice level appropriately to teach the lesson” are the same meaning, and using the average of the scores of these two items as the observation of *Volume*. The mean of these two items was used to observe *Volume* to show the subjects’ thoughts more accurately. The researchers developed a 35-item (t1-t35) five-point Likert questionnaire based on the framework. The scores for each option were recorded as 5, 4, 3, 2, and 1 in descending order of satisfaction (approval).

This study was also a scale development study. Therefore, exploratory factor analysis and confirmatory factor analysis were used to explore the suitability of the data of the structure. The McDonald’s omega coefficient was a substitute for Cronbach’s alpha and could more accurately approach the scale’s reliability (Peters, 2014). Therefore, McDonald’s omega coefficient was used in this study.

### 4.2. Participants

The participants were 316 students from a key middle school in Changsha, Hunan Province, China. They came from three grades of senior high school. First of all, according to the purpose of the study, the researchers selected a novice, skilled, and expert mathematics teacher (a total of 9 mathematics teachers) from the first, second, and third grades of the senior high school and distributed questionnaires to the students in their class. Because there are no novice mathematics teachers in the third grade of senior high school, the researchers replaced the novice mathematics teachers in the third grade of senior high school with the novice mathematics teachers in the first year of senior high school. Table 2 demonstrates the professional titles and years of teaching experience of the teachers.

**Table 2:** Mathematics teacher information.

Teacher	Title	Teaching years
Novice	None	1
Novice	Junior	2
Novice	Junior	3
Skilled	Intermediate	8
Skilled	Intermediate	9
Skilled	Intermediate	8
Expert	Senior	21
Expert	Senior	35
Expert	Senior	37

### 4.3. Procedure

This study was divided into five steps. In the first step, the researchers proposed a preliminary research question based on reflection on teaching practice, conducted a literature search and expert consultation, and determined the starting point of this study. In the second step, the researchers clearly proposed a research question based on theoretical research and practical needs, developed a research framework for the research question combined with existing research results, and revised and justified the research framework. In the third step, the researchers designed a questionnaire based on the research framework and conducted expert validation of the validity of the questionnaire. In the fourth step, the researchers recruited the participants according to the research plan, administered the questionnaire to the participants, collected the questionnaire, and transcribed the data. In the fifth step, the researchers analyzed the data, presented the results, and discussed and reflected on them concerning the existing relevant literature, pointing out the limitations of the study and research recommendations.

### 4.4. Data collection and analyzes

Data processing in this study included five processes: collection, transcription, processing, description, and analysis. For data collection, a questionnaire survey was adopted in this study. The researchers recruited potential participants, issued paper questionnaires, guided them to fill in the questionnaires as required, and collected the questionnaires. For the data transcription, after the quality of the paper questionnaires was preliminarily reviewed, invalid questionnaires were eliminated (those with missing values and those with all the same options were regarded as invalid questionnaires), and the data of valid questionnaires were input into EXCEL and SPSS 22.0. For data description, the researchers conducted descriptive statistical analysis on the primary attributes of all participants and presented preliminary descriptive statistical results. For data analysis, the researchers used exploratory factor analysis and confirmatory factor analysis to explore the suitability of the data of the structure. First, SPSS 22.0 were used to test the reliability and validity of the questionnaire. The researchers present evidence of the model’s convergence and discriminant validity and calculate and report the average variance extracted and composite reliability values (Fornell and Larcker, 1981). Then, AMOS 22.0 was used for confirmatory factor analysis, and the fit of the measurement model was discussed under the maximum likelihood method. Based on the path coefficient significance test, the model was modified and verified by referring to the ideal value of the structural equation model. The final analysis model of high school mathematics teachers’ satisfaction with audible teaching language was obtained. Finally, the paper presented the descriptive statistical results of high school students’ satisfaction with the audible teaching language of the expert, skilled, and novice mathematics teachers and conducted the difference test.

## 5. Results

A total of 316 questionnaires were distributed, 291 were returned, and 263 valid questionnaires were obtained after excluding the missing values and selecting all the same options, with an effective rate of 90.4%. The following exploratory factor analysis results are based on the first 131 of the 263 valid data, and confirmatory factor analysis results are based on the last 132 of the 263 valid data.

### 5.1. Exploratory factor analysis

The researchers counted the scores of 12 exogenous variables (observed variables), and the results showed that the alpha reliability coefficient of the scores of 12 exogenous variables was 0.949, and overall, the reliability of this questionnaire was high. According to the correlation matrix information in Table 3, all the correlation values in the data are greater than 0.3, so the data satisfy the primary conditions for factor analysis.

**Table 3:** Correlation matrix for the observed variables.

	S1	S2	S3	S4	S5	S6	S7	S8	S9	S10	S11	S12
S1	1.000											
S2	0.621	1.000										
S3	0.527	0.637	1.000									
S4	0.536	0.717	0.619	1.000								
S5	0.452	0.605	0.639	0.734	1.000							
S6	0.449	0.603	0.589	0.730	0.686	1.000						
S7	0.491	0.539	0.570	0.586	0.493	0.510	1.000					
S8	0.560	0.633	0.622	0.665	0.593	0.562	0.693	1.000				
S9	0.477	0.610	0.663	0.671	0.675	0.737	0.641	0.702	1.000			
S10	0.492	0.612	0.645	0.658	0.611	0.642	0.682	0.667	0.791	1.000		
S11	0.378	0.509	0.545	0.601	0.580	0.669	0.598	0.541	0.697	0.593	1.000	
S12	0.518	0.650	0.627	0.697	0.700	0.706	0.631	0.705	0.769	0.680	0.697	1.000

The validity of the 12 exogenous variables data shows that the KMO value was 0.948, *p* = 0.000 < 0.05, and Bartlett’s sphericity test was significant. Combined with the standard that the characteristic root is greater than 1, the explanation rate of the total variance, and the gravel diagram, this study extracted two factors according to the set research framework, see Table 4. The results show that the total variance is 71.849%.

**Table 4:** Total variance explained.

Component	Initial eigenvalues			Extraction sums of squared loadings		
	Total	% of variance	Cumulative %	Total	% of variance	Cumulative%
1	7.822	65.18	65.18	7.822	65.18	65.18
2	0.800	6.669	71.849	0.800	6.669	71.849
3	0.665	5.544	77.393			
4	0.453	3.773	81.167			
5	0.403	3.358	84.525			
6	0.364	3.033	87.558			
7	0.344	2.865	90.423			
8	0.311	2.595	93.018			
9	0.264	2.204	95.222			
10	0.224	1.867	97.089			
11	0.193	1.604	98.693			
12	0.157	1.307	100			

Extraction method: principal component analysis.

The factor load matrix after rotation is shown in Table 5. All variables can be divided into the *Sound* element and the *Content* element. The *Sound* elements include *Volume*, *Tone*, *Speed,* and *Quality*. The *Content* elements include *Sincerity*, *Inspiring*, *Interesting*, *Adaptability*, *Figurativeness* and *Feedback*, *Scientificity*, and *Conciseness*.

**Table 5:** Rotated component matrix.^a^

Exogenous variable	Component	
	1	2
Sincerity	0.833	
Inspiring	0.814	
Interesting	0.812	
Adaptability	0.763	
Figurativeness	0.726	
Feedback	0.690	
Scientificity	0.558	
Conciseness	0.540	
Volume		0.887
Tone		0.726
Speed		0.577
Quality		0.527

Extraction method: principal component analysis. Rotation method: Varimax with Kaiser normalization. ^a^Rotation converged in 3 iterations.

In this study, the McDonald’s Omega coefficient was used to describe the reliability of the tool. If this value is higher than 0.8, it means high reliability; if this value is between 0. 7and 0. 8, it is good; if this value is between 0. 6 and 0. 7, it means reliability is acceptable; if this value is less than 0. 6, it means that the reliability is not good. Standardized results showed that the overall McDonald’s omega coefficient was 0.951, the McDonald’s omega coefficient of sound dimension was 0.867, and the McDonald’s omega coefficient of content dimension was 0.938. These values were all greater than 0.8, indicating that the research data have high reliability. In conclusion, the 12 exogenous variables were well-suited for factor analysis, and the validity of the questionnaire was high.

### 5.2. Confirmatory factor analysis

The validated factor analysis model was fitted using the maximum likelihood method. There was no negative error variance, the factor loading was between 0.5 and 0.95, and there was no large standard error, indicating the good intrinsic quality of the model.

**Figure 1:**
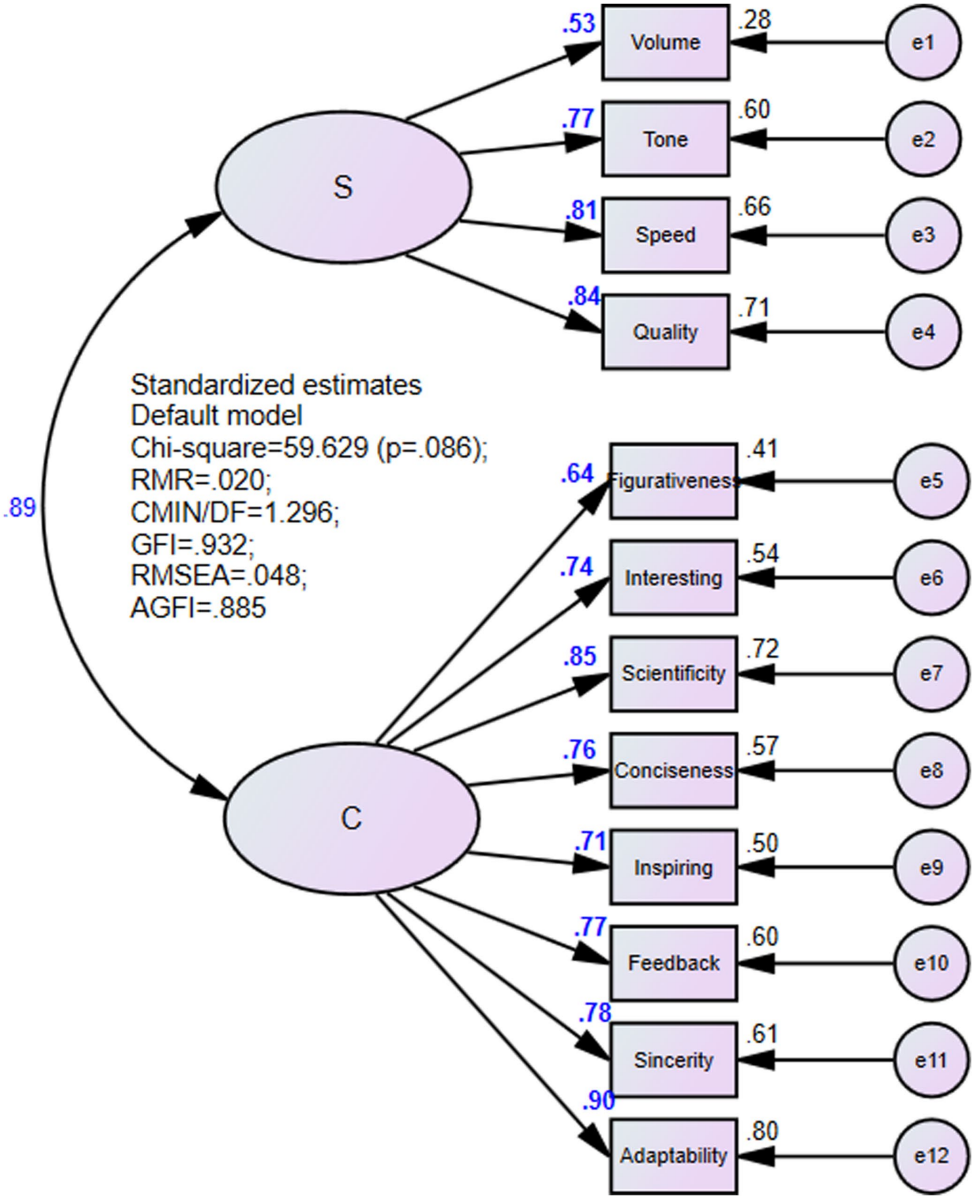
The adaptation result of the measurement model.

The results of the validated factor analysis are shown in Figure 1. The Chi-square value of the prespecified model is 59.629, with a significance probability value of 0.086 > 0.05, which does not reach the significance level and accept the null hypothesis.

In terms of model fitness statistical tests, RMR = 0.020 < 0.050, CMIN/DF = 1.296 < 2.000, AGFI = 0.885 (very close to 0.9), RMSEA = 0.048 < 0.050, GFI = 0.932 > 0.900, NFI = 0.948 > 0.900, RFI = 0.925 > 0.900, IFI = 0.988 > 0.900, TLI = 0.982 > 0.900, CFI = 0.987 > 0.900, PRATIO = 0.697 > 0.500, PNFI = 0.660 > 0.500, PCFI = 0.688 > 0.500 that meet the criteria for the model to be adaptable. The estimated parameters of the factor loadings all reach a significant level (*p* < 0.05). Therefore, the model has good fitness.

In this study, average variance extraction quantity and combinatorial reliability were used to analyze the convergence validity of the data. The average variance extracted (AVE) was more than 0.5, and the combination reliability (CR) was more than 0.60, which indicates that the results have high combination reliability and convergence validity. The average variance extracted (AVE) and combinatorial reliability (CR) of the latent variable *Sound* were 0.559 > 0.50 and 0.831 > 0.60, respectively. The average variance extracted (AVE) and combinatorial reliability (CR) of the latent variable *Content* were 0.597 > 0.50 and 0.921 > 0.60, respectively. It showed that the measurement model in this study had high combinatorial reliability and convergence validity.

In summary, the questionnaire designed in this study had good reliability and validity. It could be used to investigate high school students’ satisfaction with mathematics teachers’ audible teaching language.

### 5.3. Descriptive statistics of student satisfaction

The statistics of students’ satisfaction with mathematics teachers’ audible teaching language are shown in Table 6. From the results, it can be seen that students’ satisfaction (i.e., the sum of the percentages of basically satisfied and very satisfied) with the use of mathematics teachers’ audible teaching language is high, reaching 95.44%. Students’ satisfaction with the *Content* (95.06%) aspect of mathematics teachers’ audible teaching language was slightly higher than that of *Sound* (93.54%).

**Table 6:** Statistics of students’ total satisfaction (*N* = 263).

Satisfaction	Total		Sound		Content	
	*N*	Percentage	*N*	Percentage	*N*	Percentage
Very satisfied (4,5)	171	65.02%	165	62.74%	172	65.40%
Satisfied (3,4)	80	30.42%	81	30.80%	78	29.66%
Unsatisfied (1,3)	12	4.56%	17	6.46%	13	4.94%

As can be seen from Table 7, overall, students are the most satisfied (98.67%) with the skilled teachers’ audible teaching language, followed by the expert teachers (96.71%) and the novice teachers last (91.75%). The satisfaction received by the skilled teachers is 1.96 percentage points higher than the expert teachers, the satisfaction received by the expert teachers is 4.96 percentage points higher than the novice teachers, and the satisfaction received by the skilled teachers is 6.92 percentage points higher than the novice teachers.

**Table 7:** Students’ satisfaction with expert, skilled, and novice teachers (*N* = 263).

Satisfaction	Expert		Skilled		Novice	
	*N*	Percentage	*N*	Percentage	*N*	Percentage
Very satisfied (4,5)	67	73.63%	47	62.67%	57	58.76%
Satisfied (3,4)	21	23.08%	27	36.00%	32	32.99%
Unsatisfied (1,3)	3	3.30%	1	1.33%	8	8.25%

As can be seen from Table 8, in terms of *Sound,* students are the most satisfied with the expert teacher’s audible teaching language (95.61%), followed by the skilled teacher (93.33%) and the novice teacher last (91.76%). In terms of *Sound* of audible teaching, expert teachers receive 2.28 percentage points higher satisfaction than skilled teachers, skilled teachers receive 1.57 percentage points higher satisfaction than novice teachers, and expert teachers receive 3.85 percentage points higher satisfaction than novice teachers.

**Table 8:** Satisfaction with expert, skilled, and novice teachers in terms of *Sound* (*N* = 263).

Satisfaction	Expert		Skilled		Novice	
	*N*	Percentage	*N*	Percentage	*N*	Percentage
Very satisfied (4,5)	65	71.43%	42	56.00%	60	61.86%
Satisfied (3,4)	22	24.18%	28	37.33%	29	29.90%
Unsatisfied (1,3)	4	4.40%	5	6.67%	8	8.25%

Table 9 shows that in terms of *Content*, students’ satisfaction with audible teaching language is the highest among skilled teachers (97.33%), followed by expert teachers (96.71%) and novice teachers (90.72%).

**Table 9:** Satisfaction with expert, skilled, and novice teachers in terms of *Content* (*N* = 263).

Satisfaction	Expert		Skilled		Novice	
	*N*	Percentage	*N*	Percentage	*N*	Percentage
Very satisfied (4,5)	67	73.63%	45	60.00%	57	58.76%
Satisfied (3,4)	21	23.08%	28	37.33%	31	31.96%
Unsatisfied (1,3)	3	3.30%	2	2.67%	9	9.28%

In terms of the *Content* of audible teaching, the satisfaction of experienced teachers is 0.62 percentage points higher than that of expert teachers, 5.99 percentage points higher than that of novice teachers, and 6.61 percentage points higher than that of skilled teachers.

### 5.4. Test of variance of student satisfaction

First, the differences in the audible teaching language of expert, skilled, and novice high school mathematics teachers were analyzed with the Kruskal-Wallis test. As can be seen from the summary of hypothesis testing in Table 10 (Shows asymptotic significance with a significance level of 0.05), it is found that *p* < 0.05 in terms of *Tone* and *Adaptability* of the audible teaching language and reject the original hypothesis. It can be concluded that the differences in *Tone* and *Adaptability* of expert, skilled, and novice high school mathematics teachers are statistically significant.

**Table 10:** Kruskal-Wallis test for independent samples (expert, skilled, and novice teachers).

Original hypothesis	Sig.	Decision
The distribution of volume is the same	0.345	Retain the original hypothesis
The distribution of tone is the same	0.027	Reject the original hypothesis
The distribution of speed is the same	0.348	Retain the original hypothesis
The distribution of quality is the same	0.368	Retain the original hypothesis
The distribution of sound is the same	0.132	Retain the original hypothesis
The distribution of figurativeness is the same	0.173	Retain the original hypothesis
The distribution of interesting is the same	0.062	Retain the original hypothesis
The distribution of scientificity is the same	0.090	Retain the original hypothesis
The distribution of conciseness is the same	0.127	Retain the original hypothesis
The distribution of inspiring is the same in	0.472	Retain the original hypothesis
The distribution of feedback is the same in	0.078	Retain the original hypothesis
The distribution of sincerity is the same in	0.076	Retain the original hypothesis
The distribution of adaptability is the same	0.013	Reject the original hypothesis
The distribution of content is the same	0.137	Retain the original hypothesis
The distribution of total is the same	0.157	Retain the original hypothesis

Table 11 reflects the situation of the rank of *Tone* and *Adaptability* in the audible teaching language. In terms of *Tone*, the rank means of novice, skilled, and expert mathematics teachers are increasing in order (118.84 < 130.29 < 147.43). Thus, students’ satisfaction with the *Tone* of the audible teaching language of novice, skilled, and expert high school mathematics teachers increased sequentially. Regarding *Adaptability*, the rank means of novice, skilled, and expert mathematics teachers increase in descending order (115.80 < 135.53 < 146.53). So students’ satisfaction with the *Adaptability* of novice, skilled, and expert high school mathematics teachers’ audible teaching language increased in descending order.

**Table 11:** The rank of *Tone* and *Adaptability*.

	Group	*N*	Mean rank
Tone	1	97	118.84
	2	75	130.29
	3	91	147.43
Adaptability	1	97	115.80
	2	75	135.53
	3	91	146.35

Table 12 gives the results of the pairwise comparison of the rank means of expert, skilled, and novice high school mathematics teachers in terms of *Tone*.

**Table 12:** Paired comparison results (*Tone*).

Sample 1-Sample 2	Test statistic	Std. error	Std. test statistic	Sig.	Adj.sig
0–1	−11.453	11.278	−1.016	0.310	0.930
0–2	−28.594	10.704	−2.671	0.008	0.023
1–2	−17.141	11.439	−1.498	0.134	0.402

Each row tests the original hypothesis: sample 1 and sample 2 are equally distributed. Show asymptotic significance (2-sided test), and the significance level is 0.05.

From the results, it is clear that the rank mean of novice teachers minus the rank mean of expert teachers is−28.594 and the difference after the test is statistically significant (p < 0.05), indicating that students are more satisfied with expert teachers than with novice teachers in terms of *Tone*.

In terms of *Adaptability*, Table 13 gives the results of the pairwise comparison of the rank means of expert, skilled, and novice high school mathematics teachers. It is clear that the rank mean of novice teachers minus the rank mean of expert teachers is-30.548, and the difference after the test is statistically significant (*p* < 0.05), indicating that in terms of *Adaptability*, students are more satisfied with expert teachers than with novice teachers.

**Table 13:** Paired comparison results (*Adaptability*).

Sample1-Sample2	Test statistic	Std. error	Std. test statistic	Sig.	Adj.Sig
0–1	−19.729	11.083	−1.780	0.075	0.225
0–2	−30.548	10.519	−2.904	0.004	0.011
1–2	−10.818	11.241	−0.962	0.336	1.000

Each row tests the original hypothesis: sample 1 and sample 2 are equally distributed. Show asymptotic significance (2-sided test), and the significance level is 0.05.

## 6. Discussion and enlightenment

This study explored students’ satisfaction with the audible teaching language of high school mathematics teachers in the classroom. According to the research results, students were very satisfied with the audible teaching language of high school mathematics teachers. As the school of the participants is one of the best in the province and the professional quality of the teachers is very high, such results are expected. Overall, the students had the highest satisfaction with the skilled teachers’ audible teaching language, followed by expert and novice teachers. Some research results show that novice and skilled math teachers have no significant differences in their understanding of problem solving and teaching as a whole but significant differences in individual aspects (Jiang et al., 2022). This study showed that the above characteristics of novice and non-novice teachers also existed in the audible teaching language of mathematics teachers. Although students’ satisfaction with expert, skilled, and novice math teachers decreased successively in terms of the *Sound* of audible teaching language, and students’ satisfaction with skilled, expert, and novice math teachers also decreased successively in terms of the *Content* of audible teaching language. There was no significant difference in the overall satisfaction obtained by expert, skilled, and novice high school math teachers, there were only differences in individual observed variables. For example, in terms of *Tone* and *Adaptability*, students were significantly more satisfied with the audible teaching language of expert math teachers than novice math teachers.

Skilled teachers received higher satisfaction than expert teachers, and expert teachers received higher satisfaction than novice teachers. It is easy to understand why expert teachers outperformed novice teachers in audible teaching language, but why did skilled teachers outperform expert teachers? It may be related to the evaluation and promotion of teachers, as skilled teachers face a critical period of promotion and are under pressure from family and career, so they will try to do their best in all aspects. It also verifies the research hypothesis that proper training can improve the quality of teachers’ audio teaching language. Expert teachers, on the other hand, are under relatively less pressure. However, research findings have shown that the curriculum reform process has produced many regressive expert teachers who combine some typical novice elements and some typical expert elements in their professional practice (Liberman et al., 2012). It suggests that the motivation of expert teachers is necessary and that educational administrators should create opportunities for them to continue learning.

There was no significant difference between the expert and skilled teachers on each of the observed variables, nor was there a significant difference between skilled and novice teachers on each of the observed variables. In terms of *Tone*, there was a significant difference between expert and novice mathematics teachers, with expert teachers outperforming novice teachers. It is a significant result showing that novice mathematics teachers can improve the quality of the audible teaching language by improving the *Tone* of the audible teaching language in their teaching. In terms of *Adaptability*, expert mathematics teachers are more aware of students’ needs and are able to use appropriate audible teaching language in their teaching. It may be because expert mathematics teachers are more student-centered and not overly focused on themselves. As noted, expert teachers always respond to students by asking questions, but novice teachers point out students’ errors directly (Cai et al., 2022).

Teacher professional development is a complex, long-term process (Jin et al., 2021). Mature teachers have their own distinctive audible language styles, and these styles are not incompatible with each other, nor are they superior or inferior. In light of the above analysis, novice mathematics teachers (or pre-service mathematics teachers) should be trained to focus on the *Tone* and *Adaptability* of their audible teaching language. Pre-service teacher training programs should pay appropriate attention to the vocal aspects of instructional language. Teacher educators should provide training courses on the *Tone* and *Adaptability* of the audible teaching language so those novice mathematics teachers (or pre-service mathematics teachers) are genuinely concerned with the needs of their students, are student-centered, and teach by learning.

This study expands the knowledge in the field of teacher education and illustrates some characteristics of the audible teaching language of expert, skilled, and novice high school mathematics teachers. The study is also of great practical value, pointing out how differences should be overcome to improve the quality of the audible teaching language. However, the fact that the selected participants were from a top provincial high school, though exemplary, limits the generalization of the findings. Future studies should select a less affluent sample because the participants for this study were affluent students who attended a wealthy school.

## Data availability statement

The raw data supporting the conclusions of this article will be made available by the authors, without undue reservation.

## Ethics statement

The studies involving human participants were reviewed and approved by the Ethics Committee of Hunan Normal University. Written informed consent from the participants’ legal guardian/next of Kin was not required to participate in this study in accordance with the national legislation and the institutional requirements.

## Author contributions

PJ designed the study, developed the research tools, and wrote the paper. XZ collected data for this research. XR and ZF analyzed and processed the data. BX entirely directed this study and completed the correspondence. YJ assisted in communication correspondence and data analysis. All authors contributed to the article and approved the submitted version.

## Funding

This study was funded by a grant from the Shanghai Key Laboratory of Pure Mathematics and Mathematical Practice 18dz2271000.

## Conflict of interest

The authors declare that the research was conducted in the absence of any commercial or financial relationships that could be construed as a potential conflict of interest.

## Publisher’s note

All claims expressed in this article are solely those of the authors and do not necessarily represent those of their affiliated organizations, or those of the publisher, the editors and the reviewers. Any product that may be evaluated in this article, or claim that may be made by its manufacturer, is not guaranteed or endorsed by the publisher.
